# Falling into the trap: A study of the cognitive neural mechanisms of immediate rewards impact on consumer attitudes toward forwarding perk advertisements

**DOI:** 10.1371/journal.pone.0302023

**Published:** 2024-06-10

**Authors:** Rui Sun, Jiajia Zuo, Xue Chen, Qiuhua Zhu

**Affiliations:** School of Business Administration, Huaqiao University, Quanzhou, Fujian, China; Universidad Central de Chile, CHILE

## Abstract

In the context of digital marketing, consumers often express aversion to perk advertisements yet find it challenging to resist the temptation and forward it, resulting in inconsistent attitudes and behaviors. This study, based on the Associative Propositional Evaluation model and the Confirmation Bias theory, utilizes event-related potential experiments to identify the interactive impacts of immediate rewards and information diagnosticity in advertisements on consumer attitude change in specific contexts. The research findings indicate that when rewards were present, information diagnosticity positively influences attitude change and the willingness to forward. However, when rewards were absent, the impact of information diagnosticity on attitude change and the willingness to forward is not significant, and neuroscientific evidence supports these findings. Theoretically, this study extends the research perspective on attitude change in online advertising contexts and broadens the application of the Associative Propositional Evaluation model in the field of consumer attitude change towards advertisements. In practice, this research holds significant guiding value for constraining platform manipulation of consumer cognitive behaviors, guiding the healthy development of platform economics, and promoting digital technology ethics.

## 1. Introduction

Consumers are disgusted but still happy with the endless perk advertisements [1] that platforms are launching to achieve new customer acquisition. Examples include messages like "Congratulations, you’ve drawn the easiest mode, and inviting 10 people can earn you 300 yuan in cash!" and "Congratulations, you’ve received a 0.2 yuan in WeChat account." Consequently, the platform rapidly acquires a significant amount of traffic and enlarges its market share. However, the platform manipulates consumers’ cognition through perk advertisements, turning them into "puppets" for sharing and attracting new users [2, 3]. This constitutes an infringement on consumers’ free will and autonomy [4, 5], inevitably leading to the erosion of trust, negative platform reputation, and even resistance, all of which pose severe threats to the platform’s long-term development [6]. Therefore, investigating the intrinsic cognitive mechanisms of perk advertisements’ impact on consumers’ immediate forwarding behavior holds significant practical importance for protecting user autonomy and interests, strengthening government regulation against platform violations of business ethics, standardizing the environment for user acquisition marketing on platforms, guiding the healthy development of platform economics, and promoting the ethical advancement of digital technology.

Scholars have conducted extensive and valuable research on the generation and alteration of consumer attitudes toward advertising, recognizing its importance for digital marketing research. In an online context, consumer attitudes toward advertising influence online information retrieval behavior [7], positively impact brand attachment [8], foster more favorable attitudes toward brands [9], enhance willingness to revisit and brand trust [10–12], increase purchase intention [13], prompt impulse buying decisions [14], create positive electronic word-of-mouth [15, 16], and promote eco-friendly consumption [17, 18], among other effects. Despite exploring the antecedents and mechanisms of consumer advertising attitude formation and change in online marketing contexts from different theoretical perspectives, these research findings have mainly focused on the characteristics of traditional advertising in mass media [19–21]. Consequently, existing research have limitations in explaining fully the impact of rapidly iterated, precise perk advertisements empowered by intelligent algorithms on consumer attitudes. Additionally, theories based on relatively stable attitudes and research approaches that separate attitudes from behaviors cannot adequately address the requirement for real-time attitude changes by consumers in online instantaneous contexts [22]. Some gaps in the existing research are as follows:

Firstly, traditional advertising marketing is generic advertising aimed at a homogenous audience, gradually changing attitudes through continuous exposure [23], rational appeals [24], or emotional appeals [25]. In contrast, perk advertisements are rapidly changing consumer attitudes by target messages efficiently through algorithms, prompting immediate decisions [26]. Additionally, perk advertisements, unlike traditional static advertising, exhibit iterative variations, leading to a mismatch between research conclusions on traditional ad attitudes and the new context.

Secondly, previous research on consumer attitudes toward static advertising has explored factors such as motivation [27, 28] and cognitive processing styles [29]. These studies have adopted theoretical perspectives like the Persuasion Knowledge Model [30–32], the Elaboration Likelihood Model [33, 34], and the Dual-System Model [35]. They argue that attitudes influencing behavior are stable and formed through single cognitive processing of advertising information. In contrast, perk advertisements differ from static advertising as they carry evolving new information due to algorithmic upgrades. Consequently, consumer attitude formation and change regarding perk advertisements is a dynamic process. This renders the perspective of relatively stable advertising attitudes inadequate to fully explain shifts in consumer attitudes toward perk advertisements.

Thirdly, situational experiments have been the primary method to investigate changes in consumer advertising attitudes. Typically, consumers are asked to report their behavioral inclinations and attitudes after advertising stimuli. This research approach is not suitable for capturing immediate attitude changes in online contexts. Instant attitude changes can be spontaneous processes occurring at the subconscious level, making it challenging for consumers to consciously perceive and accurately report them [36]. Moreover, advertising information, behavioral intentions, and advertising attitudes are discrete and lack continuity [37], relying on hypothetical causal responses within assumed scenarios. Additionally, relying on consumers’ retrospective analyses of processed measurement data is not authentic [38]. Together, these limitations mean a lack of direct real-time objective evidence for the genuine attitude change process of consumers in the instant forwarding decision of perk advertisements, rendering the cognitive black box unopened [37].

To address these limitations, this study utilizes Event-Related Potentials (ERPs) experiments combined with self-report methods. Based on the Associative Propositional Evaluation Model (APE Model) and Confirmation Bias Theory, we investigate how immediate emotions evoked by advertising stimuli interact with immediate proposition evaluation to influence consumer attitudes and forwarding behavior toward perk advertisements. ERPs, with their high temporal resolution, can capture real-time processes that are beyond the scope of individual consciousness. This approach delineates the cognitive processing involved in individual attitude changes within the immediate stimulus-response pattern in real-time situations, providing accurate and objective insights into the interaction mechanisms between immediate rewards and diagnostic information in shaping consumer attitudes and forwarding behavior toward perk advertisements.

## 2. Literature review and research hypotheses

### 2.1 Research on consumer advertising attitude formation and change

Previous research on consumer attitudes towards advertising has undergone extensive and beneficial exploration. Numerous studies applied the Elaboration Likelihood Model, which proposed that persuasive knowledge activation can resist persuasion and inhibit changes in advertising attitudes [32]. Some studies also employed the Central Route and Peripheral Route of the Elaboration Likelihood Model to explain the formation and changes in advertising attitudes. Scholars explored the interaction between involvement and advertising appeal types in advertising attitude changes [39]. The Dual-Process Model is also frequently used to explain consumer advertising attitudes, with consumers in forming attitudes based on intuitive associations of emotional appeals in the heuristic route and forming attitudes through deep-level processing of rational appeals in the systematic route [40]. Research has also focused on the significant role of initial attitudes in advertising attitude changes, suggesting that reporting positive (compared to neutral) initial attitudes results in higher levels of positive attitude change [41]. Furthermore, to explain the inconsistency between consumer attitudes and behavior, research primarily focused on two directions. One direction has shifted toward the Dual Attitude Model, examining the impact of implicit attitudes and explicit attitudes on behavior [42], and has started to investigate the effects of subliminal stimuli [43], incidental exposure [44, 45], and other effects on the subconscious attitude formation of consumers in advertising [9]. The other direction focuses on attitude accessibility [46, 47], attitude confidence [48–50], attitude strength [51, 52], and metacognitive dimensions, exploring their predictive roles in behavior [53].

To address the dual attitudes and dual processes in advertising attitudes, scholars gradually introduced the Associative-Propositional Evaluation Model into the field of advertising marketing. They have explored the impact of humorous advertisements on both positive and negative brand associations, stimulating two processes that lead to spontaneous brand choice. Research indicates that humor, during the cognitive process, utilizes limited cognitive resources by diverting attention, thereby inhibiting the formation of negative brand associations. In the emotional process, humor triggers positive emotions, thus creating positive brand associations [54].

### 2.2 Associative-Propositional Evaluation Model

The Associative-Propositional Evaluation Model (APE Model) posits that attitudes are constructed in the moment, influenced by two processes: the associative process of memory-based mental associations activated during attitudes and the propositional process of judgment based on deductive reasoning [55]. The associative process determines which mental content is automatically activated in response to an object, primarily influenced by the similarity between input stimuli and existing representation features, as well as contextual effects. The activation of related positive or negative concepts automatically triggers emotional responses consistent with the valence of those concepts, without assessing their truth value [56].

The propositional process involves the validation of propositions transformed from spontaneous emotional responses during the associative process, based on the principle of cognitive consistency [57]. When a proposition is inconsistent with temporarily considered propositions, individuals, to avoid the discomfort of cognitive dissonance [58], will reassess the effectiveness of each component and either reject one of the relevant propositions (i.e., reversing the subjective truth value of that proposition) or seek additional propositions to resolve the inconsistency, thus restoring consistency [59].

Consumer attitudes toward perk advertisements are generated by consumers in specific contexts based on situational information. This process includes not only the conscious propositional evaluation process activated by persuasive knowledge activation but also the automatic emotional response associative process triggered by immediate monetary rewards. The combined effects of these processes shape consumers’ immediate attitudes and behaviors toward perk advertisement.

### 2.3 Confirmation bias theory

Confirmation bias refers to individuals seeking or interpreting information in a way that aligns with their existing beliefs, expectations, or hypotheses [60]. Once an attitude tendency is formed, individuals’ cognitive systems assign greater weight to information that favors their choices, beliefs, or assumptions relative to information opposing them [61–63]. Reinterpreting information individuals encounter or selecting information consistent with existing beliefs can reinforce those beliefs to achieve cognitive equilibrium and avoid the discomfort of cognitive dissonance [64].

When perk advertisement triggers a positive automatic emotional response, consumers develop an attitude tendency to share such advertising. To maintain this tendency and resolve conflicts with negative propositions formed by persuasive knowledge activation, consumers may engage in biased interpretation of diagnostic information to achieve cognitive consistency.

### 2.4 Hypothesis development

In the previous interactions with perk advertisement, consumers repeatedly expended a great deal of effort and time to share content in the hope of meeting the minimum sharing threshold to obtain substantial incentives. The process of multiple attempts and failures serves as a process of propositional learning, whereby consumers generate negative propositional inferences about perk advertisement based on their repeated unsuccessful experiences. After verifying these propositions through the process of propositional evaluation, new mental associations are created in memory, forming negative attitudes toward perk advertisement. This constitutes persuasive knowledge about perk advertisement [32, 56]. When perk advertisement does not offer an immediate reward for sharing, consumers exposed to the advertising stimulus experience the top-down activation of their negative attitude proposition about perk advertisement [65]. This activation triggers more prospective negative emotions consistent with that negative attitude, intensifying consumers’ negative attitude toward perk advertisement. As a result, consumers are inclined not to forward. Information diagnosticity, serving as a cue for consumers to judge whether they can obtain allowance, is influenced by confirmation bias. Individuals tend to generate explanations consistent with their current attitude tendency [66], during top-down persuasive knowledge activation. Through this process, the level of information diagnosticity is not a significant factor influencing sharing intention. Furthermore, individuals’ negative attitude toward perk advertisement remains unchanged. Based on this, we propose the following hypotheses:

H1: In the absence of immediate rewards, the information diagnosticity does not significantly influence forwarding intention.
H2: In the absence of immediate rewards, the information diagnosticity does not significantly influence attitude change.

The APE model suggests that the associative process involves the activation of stimulus-related concepts, and this process does not involve judgments about the validity of the implied relationships in the activated links [56]. The attainment of rewards is typically accompanied by emotional reactions. When individuals receive immediate rewards, it activates positive emotions such as joy, pleasure, and satisfaction, influencing their emotional state, motivation, and decision-making [67]. When consumers share perk advertisement and receive immediate rewards, the associative process is automatically activated. The brain circuits associated with rewards are also automatically activated, leading to the experience of positive emotions such as joy and satisfaction. The emotional reactions generated by concepts activated through association are transformed into propositional statements, which provide input for propositional reasoning, thereby influencing individuals’ propositional processes from the bottom up [56]. Propositions about consumers’ negative attitudes toward perk advertisement are inconsistent with the propositional statements about positive attitudes generated by the associative process. The new propositions formed by the immediate rewards have a higher valence compared to the propositions about the negative attitudes toward perk advertisement. Under the motivation of immediate positive emotions, individuals engage in directed memory search to retrieve positive information about sharing perk advertisement [68, 69]. Through this process, diagnostic information about allowance is used as a reliable source of cues and enters the fine-grained propositional process. High-diagnosticity information has clear and detailed meanings, providing individuals with detailed information to judge the attainability of allowance. In contrast, low-diagnosticity information is vague and ambiguous, making it difficult for individuals to judge the attainability of allowance [70]. Therefore, compared to low-diagnosticity information, high-diagnosticity information motivates individuals more to forward, thereby promoting attitude change and sharing behavior.

H3: In the presence of immediate rewards, information diagnosticity positively influences forwarding intention.
H4: In the presence of immediate rewards, information diagnosticity positively influences attitude change.

P2 is a positive component of ERP that occurs in the early unconscious attention phase of visual stimulus cognitive processing decisions. Its latency generally appears around 200ms after stimulus presentation, reflecting the automatic and rapid evaluation of stimuli. The greater the uncertainty of the stimuli, the larger the P2 amplitude [71]. In studies related to monetary rewards, P2 is associated with reward processing and attention allocation. A larger P2 amplitude indicates that individuals allocate more attentional resources to the stimuli [72]. Immediate rewards lead to more attentional resource allocation and positive emotions. Individuals are motivated to assess the uncertainty of allowance. The higher the information diagnosticity, the lower the uncertainty, leading to a smaller P2 amplitude. However, if no rewards are offered, the activation of negative attitudes toward perk advertisement causes individuals to ignore diagnostic information about allowance. This results in no significant difference in the impact of information diagnosticity on attention allocation.

H5: In the absence of immediate rewards, information diagnosticity does not significantly affect P2 amplitude.
H6: In the presence of immediate rewards, information diagnosticity negatively affects P2 amplitude.

The N2 component is a negative voltage component with a latency of around 250-330ms. The amplitude of the N2 component can represent the degree of cognitive conflict in individuals, with larger conflicts leading to larger N2 amplitudes [73]. Consumers do not have strong prior beliefs about perk advertisement. When there are immediate rewards, the associative process generates propositional statements of positive emotional tendencies toward perk advertisement. In order to maintain the stability and dominance of this attitude, individuals introduce new propositions to resolve inconsistencies with persuasive knowledge [59]. Individuals process information about allowance, where high-diagnosticity information provides specific information about the attainability of allowance. Individuals realize that obtaining allowance is challenging, and they need to balance the positive attitude generated during the associative process with their initial negative attitude, resulting in greater cognitive conflict. Low-diagnosticity information provides less information about reward attainability, motivating individuals to engage in confirmation bias cognitive processing to maintain a positive attitude [63]. In this case, cognitive conflict is smaller, resulting in a smaller N2 amplitude. When perk advertisement provides no immediate reward, persuasive knowledge is activated, and individuals’ negative attitudes toward perk advertisement are relatively stable. Diagnostic information about allowance is evaluated as information that maintains negative attitudes, leading to no significant impact of information diagnosticity on cognitive conflict.

H7: In the absence of immediate rewards, information diagnosticity does not significantly affect N2 amplitude.
H8: In the presence of immediate rewards, information diagnosticity positively affects N2 amplitude.

## 3. Methods

The experiment adopted a within-subject design of 2 (immediate reward: yes vs. no) * 2 (information diagnosticity: high vs. low) to investigate their interactive effects on users’ forwarding behavior and attitude change towards perk advertisement. Each participant went through all four levels of experimental treatments, including immediate reward with high information diagnosticity, immediate reward with low information diagnosticity, no immediate reward with high information diagnosticity, and no immediate reward with low information diagnosticity. Meanwhile, to eliminate the interference of order effects, we randomized the order of experimental treatments experienced by the participants in the experiment. The advantage of a within-subject design adopted in this study is that it can eliminate result biases caused by individual differences, making statistical tests more sensitive. In experimental design, we controlled for unrelated variables other than the presence or absence of immediate reward and information diagnosticity.

### 3.1 Participants

A recent systematic review on the applicability of electroencephalography in marketing research reported that prior studies used on average sample sizes between 16 to 42 [74, 75]. In April-May 2023, we recruited 22 students from a university in southern China who had repeatedly participated in perk advertisement forwarding activities and held negative attitudes towards such advertising in order to participate in this experiment for economic rewards. Among them, 10 were female, with an average age of 23.9 years, right-handed, and had normal unaided or corrected vision, with no history of physical or mental illness. All participants would receive a remuneration of 50–80 yuan after the experiment. The study is approved by Huaqiao University Medical Ethics Committee (Ethical research No.M2023009). All participants signed a written informed consent form before the experiment.

### 3.2 Experimental materials

**Fig 1:**
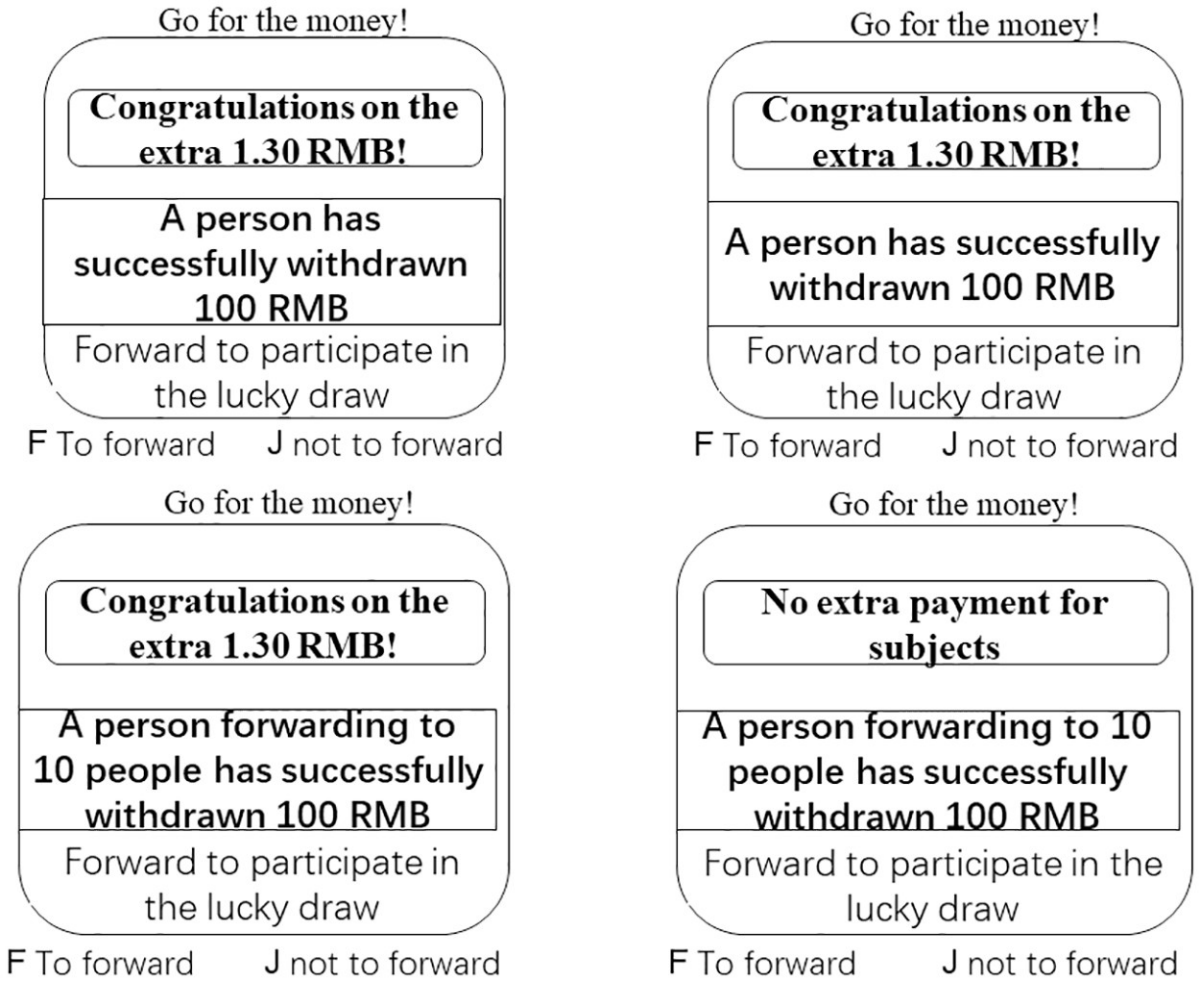
Examples of stimulus materials.

The study emulated the characteristics of perk platform advertising to develop a laboratory perk advertisement marketing campaign. Participants were invited to forward perk advertisements to assist the laboratory in recruiting participants. Immediate reward was manipulated by whether participants received additional participant fees, while information diagnosticity was manipulated by the level of detail in the estimated difficulty of obtaining allowance. To avoid personal names influencing results, names in the experimental materials were presented in alphabetical form, as illustrated in Fig 1.

### 3.3 Experimental procedure

Prior to the ERPs experiment, participants were invited to complete a questionnaire measuring their attitudes towards perk advertisement, serving as an initial attitude measurement. Additionally, the process of completing the attitude questionnaire elevated the accessibility of persuasive knowledge. The first stage of measuring attitudes towards perk advertisement took approximately 3–5 minutes. After completing the measurement, participants proceeded to the ERPs experiment. This experiment lasted approximately 90 minutes on average, including set-up time.

Participants were provided an instruction that “Imagine you are participating in a laboratory subject recruitment activity with a perk advertisement named “Go for the money!”. If you forward this perk advertisement to others to invite them to participate in research, you will have chance to get money.” After participants understanded the experiment instructions, they entered the practice phase to ensure understanding the experimental scenario and familiarize with the task. The experiment consisted of 170 total trials, with 10 practice and 160 experimental divided into 4 blocks, each containing 40 trials. Participants rested for 5 minutes between each block. Participants were again invited to complete a questionnaire measuring their attitudes towards perk advertisement in this rest period. The order of the block appeared was randomized, and the order of trials in each block was randomized.

**Fig 2:**
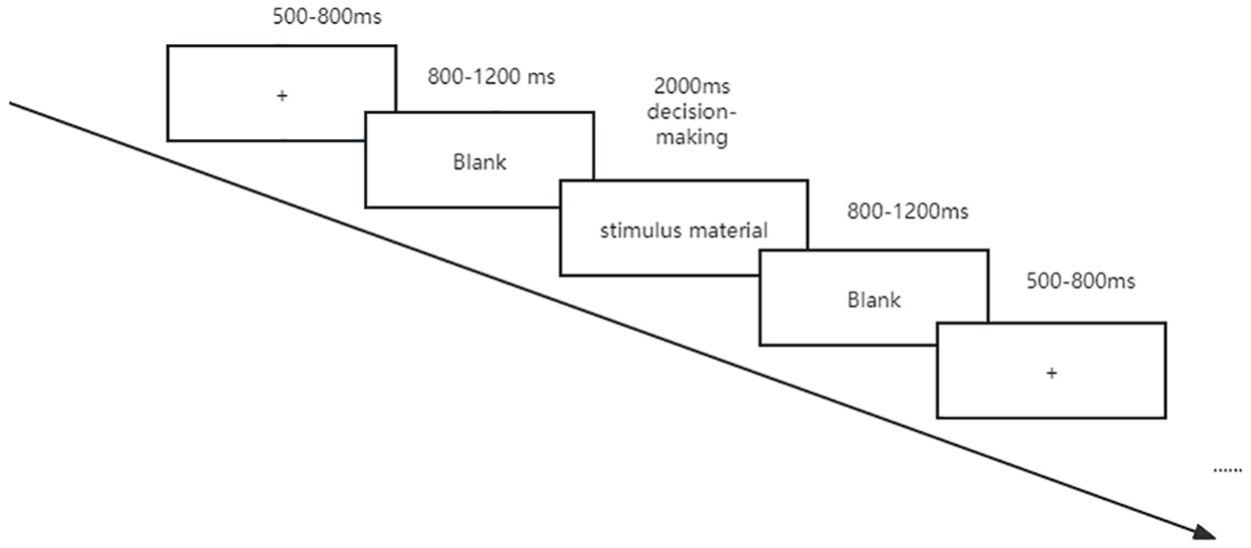
Experimental procedure.

The procedure for a single trial was as follows: a fixation point was presented for 500–800 milliseconds, followed by a blank screen for 800–1200 milliseconds. Subsequently, the experimental stimulus material was displayed. The stimulus material was a perk advertisement named “Go for the money!” with different immediate reward and diagnosticity information, as shown in Fig 1. And participants were required to read the advertisement and make decision weather forward the perk advertisement to participate in the lucky draw for more money by key press, with the "F" key representing "to forward" and the "J" key representing "not to forward" in 2000 milliseconds. After an interval with a random duration of 800–1200 milliseconds, the single trial procedure ended, and this was repeated for each trial until the experiment concluded, as illustrated in Fig 2.

The attitude measurement was adapted from established scales [76], with nine items: I consider forwarding perk advertisement to be unwise. I believe forwarding perk advertisement is a valueless and meaningless activity, a marketing trap. I believe forwarding perk advertisement is deceitful, and I cannot obtain any rewards. I dislike the marketing approach of perk advertisement. I am not interested in perk advertisement. Perk advertisement cannot attract me. I am unwilling to forward perk advertisement again. I will not continue to participate in perk advertisement marketing activities in the future. I will not invite friends around me to forward and support.

### 3.4 EEG data collection and preprocessing

In this experiment, EEG data were recorded using a 64-channel Ag/AgCl electrode cap and the Neuroscan Synamp2 Amplifier (Curry 7, Neurosoft Labs, Inc.. USA). E-prime 2.0 was used to record the key response of forwarding decisions. The electrode positions followed the international standard 10–20 system. The electrode impedance was maintained below 10 kΩ, the band-pass continuous recording EEG data was set at 0.05–100 Hz, and the sampling rate was 1000 Hz. Bilateral mastoid M1 and M2 processes served as the reference electrode. The data applied baseline correction, eye movement correction, artifact removal, and removal of data with wave amplitudes exceeding ±100μV. EEG recordings were then digitally filtered with a 30 Hz low-pass filter. Each EEG period was corrected for baseline from 200ms before stimulation to 800ms after stimulation onset, using the 200ms before stimulation onset as the baseline. The EEG data were superimposed and averaged to obtain ERPs data. Finally, the peak value was extracted separately for each stimulus type.

P2 and N2 were analyzed. For the P2 component, electrodes P1, P2, PO3, POZ, and PO4 in the left and right parietooccipital regions were selected for analysis within the time window of 150-250ms. For the N2 component, electrodes FCZ, C1, CZ, C2, and CPZ in the frontal-central region were selected for analysis within the time window of 250-350ms.

### 3.5 Results and analysis

#### (1) ERPs data analysis

**Fig 3:**
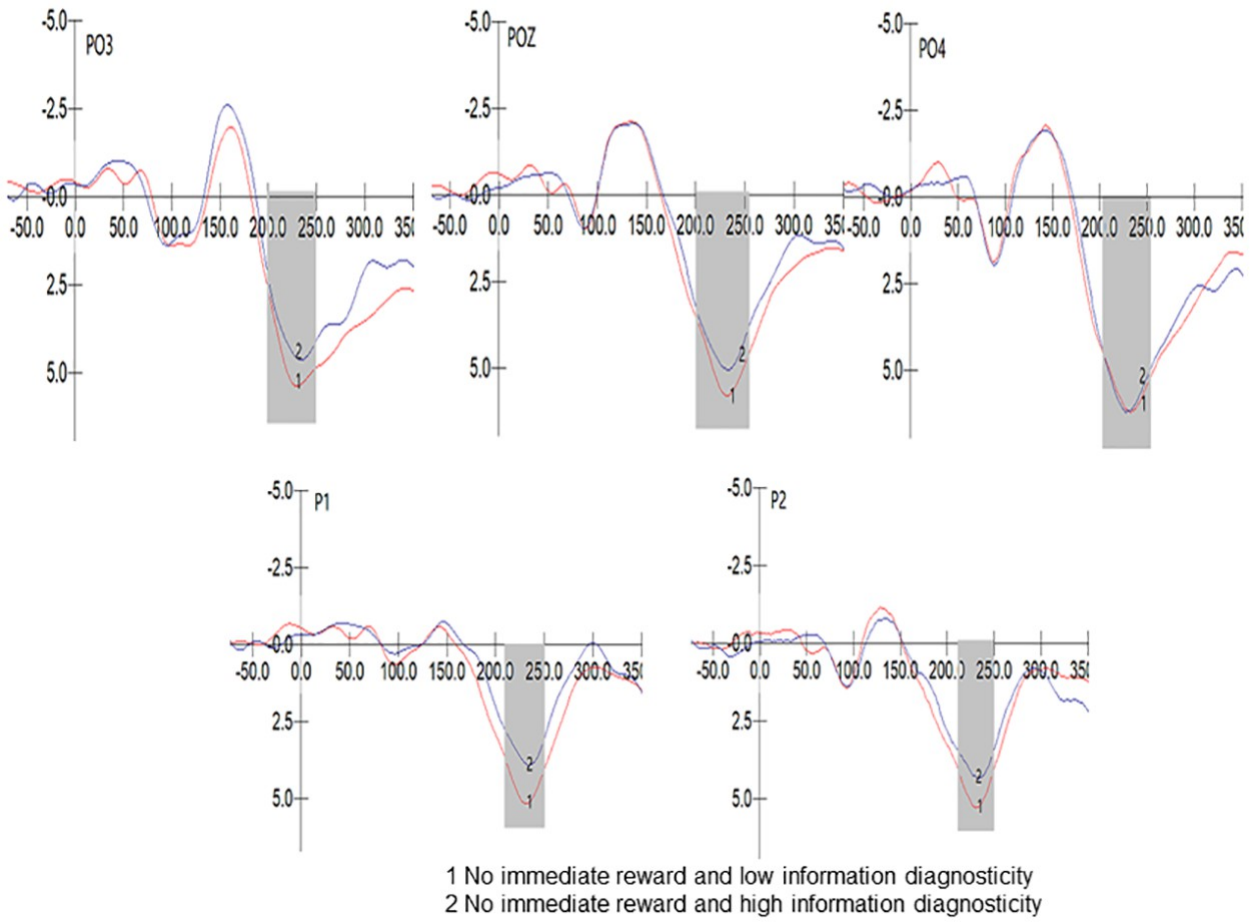
P2 component of no immediate reward group.

We used SPSS 23.0 to process the data and verify all assumptions. A repeated measures analysis of variance (ANOVA) was conducted on the P2 component peak values in the no immediate reward group with a 2 (diagnosticity: high/low) * 5 (electrode sites: P1/P2/PO3/POZ/PO4) design. The results showed that Mauchly’s test of sphericity yielded p < 0.001, indicating a violation of the sphericity assumption. The multivariate test revealed a significant main effect of electrode site, F (4, 39) = 7.795, p < 0.001, η2 p = 0.444. However, there was no significant interaction effect between electrode site and diagnosticity, F (4, 39) = 1.152, p = 0.347 > 0.05, η2 p = 0.106. The main effect of diagnosticity was also not significant, F (1, 42) = 1.601, p = 0.213 > 0.05; η2 p = 0.037. The partial eta squared was 0.037, indicating a small effect size. Therefore, the impact of diagnosticity on P2 amplitude was not significant (M _High_ = 5.173, M _Low_ = 6.063), supporting H5. The P2 component in the no immediate reward group is depicted in Fig 3.

**Fig 4:**
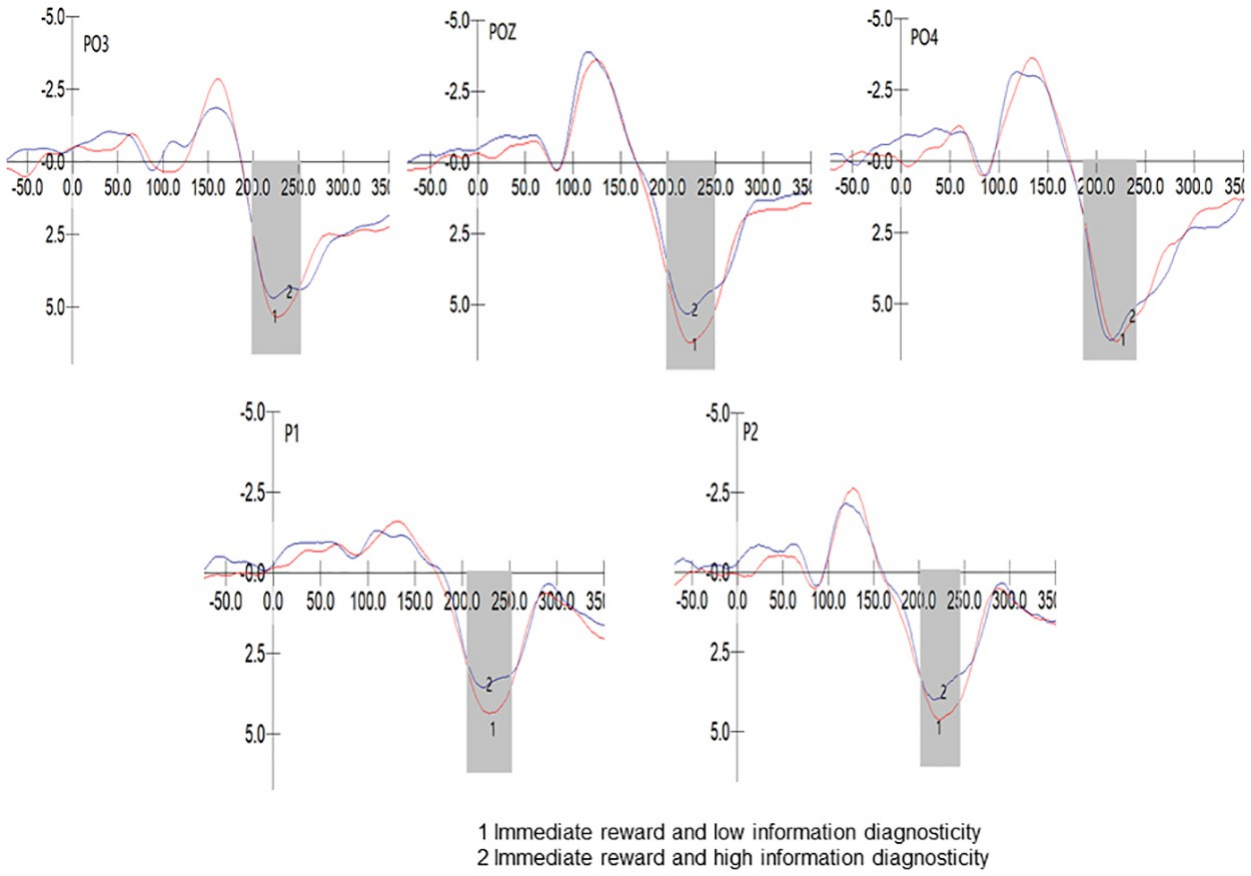
P2 component of immediate reward group.

We conducted a 2 (Diagnosticity: High/Low) * 5 (Electrode Sites: P1/P2/PO3/POZ/PO4) repeated measures ANOVA on the P2 component peak values in the immediate reward group. The results showed that Mauchly’s test of sphericity yielded p<0.001, indicating a violation of the sphericity assumption. The multivariate test indicated a significant main effect of electrode sites, F (4, 39) = 15.321, p<0.001, η2 p = 0.611; and no significant interaction effect between electrode sites and diagnosticity, F (4, 39) = 0.249, p = 0.909>0.05, η2 p = 0.025. The diagnosticity effect was significant, F (1, 42) = 4.602, p = 0.038<0.05, η2 p = 0.099, with higher diagnosticity associated with smaller P2 amplitudes (M _High_ = 4.661, M _Low_ = 6.5904). The partial eta squared was 0.099, indicating a medium effect size. Thus, Hypothesis H6 was supported. The waveform of the P2 component in the immediate reward group is shown in Fig 4.

**Fig 5:**
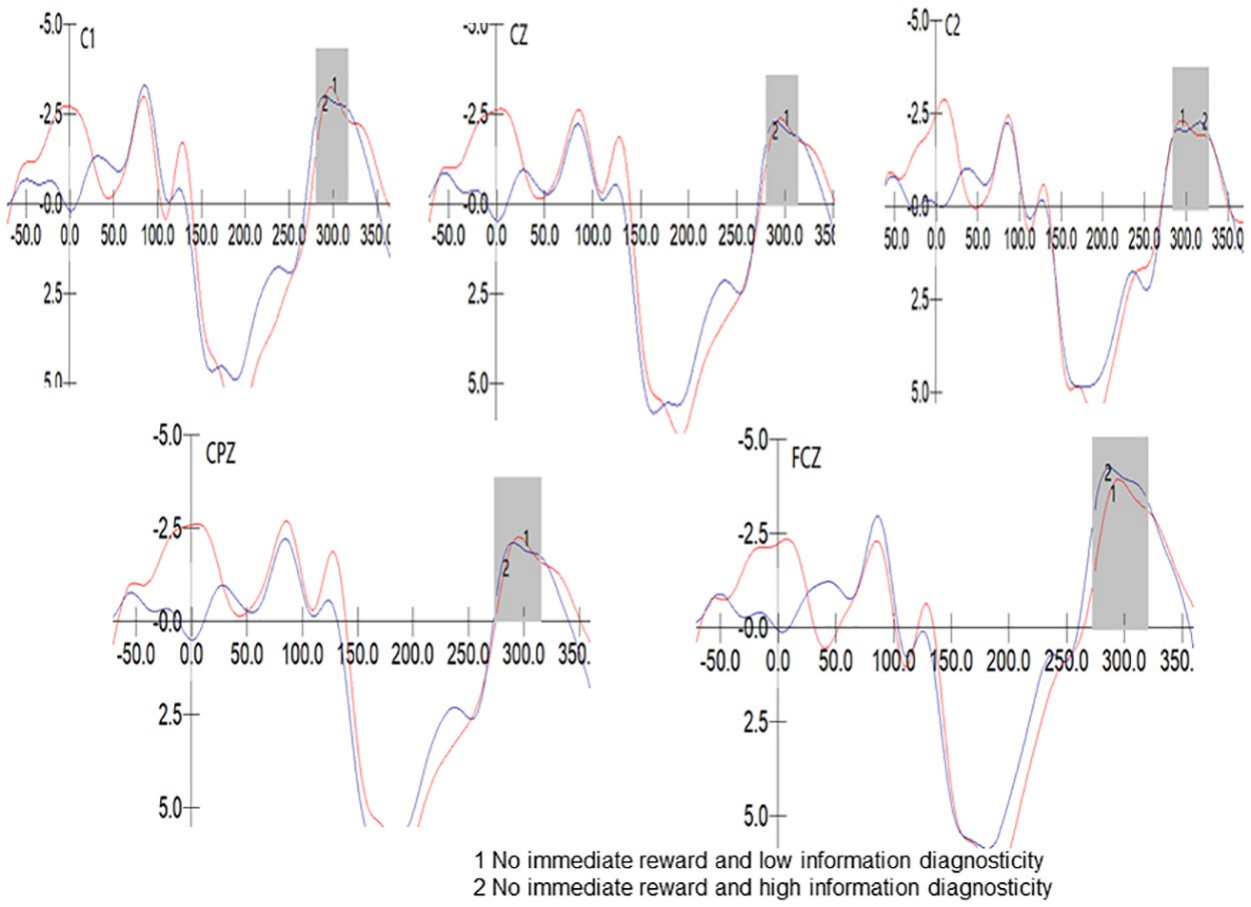
N2 component of no immediate reward group.

Conducted a 2 (Diagnosticity: High/Low) * 5 (Electrode Sites: FCZ/C1/CZ/C2/CPZ) repeated measures ANOVA on the N2 component peak values in the no immediate reward group. The results showed that Mauchly’s test of sphericity yielded p<0.001, indicating a violation of the sphericity assumption. The multivariate test indicated a significant main effect of electrode sites, F (4, 39) = 19.445, p<0.001, η2 p = 0.666; and no significant interaction effect between electrode sites and diagnosticity, F (4, 39) = 0.427, p = 0.788>0.05, η2 p = 0.042. Moreover, the diagnosticity effect was not significant, F (1, 42) = 3.277, p = 0.077>0.05, η2 p = 0.072. with no significant impact of diagnosticity on N2 amplitude (M _High_ = -4.361, M _Low_ = -3.055), supporting H7. The waveform of the N2 component in the no immediate reward group is depicted in Fig 5.

**Fig 6:**
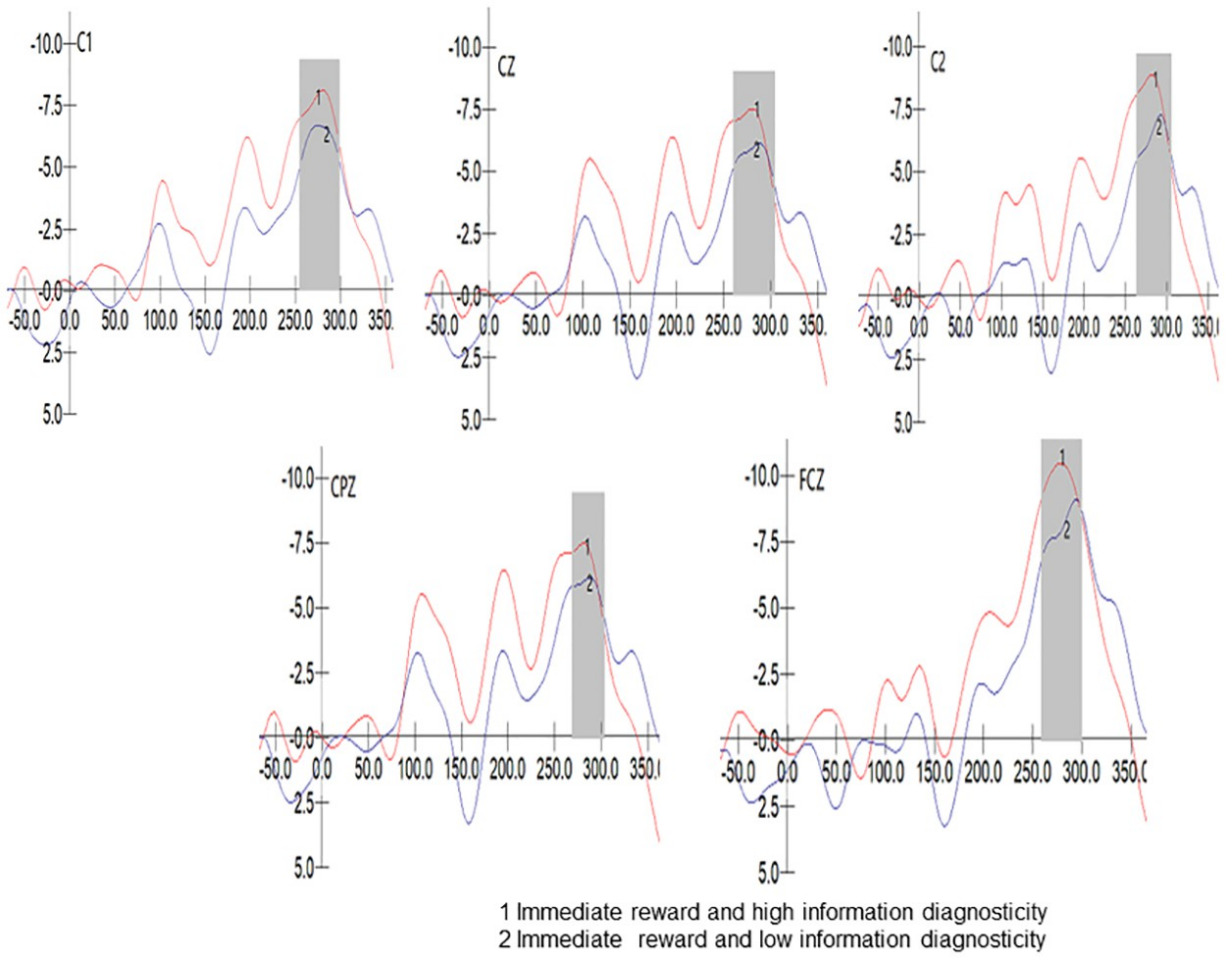
N2 component of immediate reward group.

We conducted a 2 (Diagnosticity: High/Low) * 5 (Electrode Sites: FCZ/C1/CZ/C2/CPZ) repeated measures ANOVA on the N2 component peak values in the immediate reward group. The results indicated that Mauchly’s test of sphericity yielded p<0.001, indicating a violation of the sphericity assumption. The multivariate test revealed a significant main effect of electrode sites, F (4, 39) = 17.754, p <0.001, η2 p = 0.646, while the interaction effect between electrode sites and diagnosticity was not significant, F (4, 39) = 1.401, p = 0.252>0.05, η2 p = 0.126. However, the diagnosticity effect was significant, F (1, 42) = 4.515, P = 0.040<0.05, η2 p = 0.097, with higher diagnosticity associated with larger N2 amplitudes (M _High_ = -4.812, M _Low_ = -3.408). The partial eta squared was 0.097, indicating a medium effect size. Therefore, Hypothesis H8 was supported. The waveform of the N2 component in the immediate reward group is displayed in Fig 6.

#### (2) Behavioral data analysis

An independent samples t-test was conducted on the forwarding rate in the no immediate reward group. The results showed that there was no significant difference between the high diagnosticity condition (M _High_ = 0.190, SD = 0.18) and the low diagnosticity condition (M _Low_ = 0.178, SD = 0.215), t (42) = 0.187, p = 0.853>0.05, Cohen’s d = 0.057. Hypothesis H1 was supported.

An independent samples t-test was conducted on the forwarding rate in the immediate reward group. The results showed that the forwarding rate was higher in the high diagnosticity condition (M _High_ = 0.578, SD = 0.249) than in the low diagnosticity condition (M _Low_ = 0.300, SD = 0.186), t (42) = 4.236, p < 0.001, Cohen’s d = 1.277. Hypothesis H3 was supported. Cohen’s d is a common metric of effect size with suggested interpretations as follows: 0.8 is a large effect, 0.5 is a medium effect, and 0.2 is a small effect [77]. The effect size was 1.277, indicating that information diagnosticity had a large effect on the forwarding rate.

#### (3) Self-reported data analysis

The degree of attitude change was represented by the difference between post-test attitude and pre-test attitude. Independent sample t-tests were conducted on the degree of attitude change to explore the impact of experimental treatments on participants’ attitudes towards forwarding perk advertisement.

An independent samples t-test was conducted on the degree of attitude change. The results showed that in the absence of immediate rewards, there was no significant difference in the degree of attitude change between high diagnosticity (M _High_ = 0.076, SD = 0.592) and low diagnosticity (M _Low_ = 0.040, SD = 0.593), t(42) = 0.198, p = 0.844>0.05, Cohen’s d = 0.060. Hypothesis H2 was supported.

When immediate rewards were present, the degree of attitude change was much higher in high- diagnosticity condition (M _High_ = 1.652, SD = 0.997) than in low-diagnosticity condition (M _Low_ = 0.935, SD = 0.909), t(42) = 2.493, p = 0.017<0.05, Cohen’s d = 0.752. Hypothesis H4 was supported. The effect size was 0.752, indicating that information diagnosticity had a large effect on the change of consumers’ attitude towards perk advertisements.

## 4. Discussion and conclusion

This study provides a new explanation for real-time attitude change in online perk advertisements based on the real-world phenomenon of consumers holding negative attitudes towards such advertisements but being repeatedly persuaded to share them. This experiment measured the joint impact of immediate reward and information diagnosticity on consumers’ attitudes towards perk advertisements in two stages before and after, revealing the potential mechanism of how consumers with initial negative attitudes change their existing views and share the advertisements through ERPs components. The results showed that immediate reward and information diagnosticity interactively affect consumers’ forwarding behavior and attitude change. With immediate reward, high information diagnosticity elicited more forwarding intention and attitude change compared to low information diagnosticity. In addition, information diagnosticity had a large effect on the change of consumers’ attitude towards perk advertisements and forwarding intention. Without immediate reward, information diagnosticity did not significantly influence forwarding intention.

Immediate reward, as a crucial factor affecting consumers’ emotions and motivational tendencies, activated reward-related brain areas during the attitude construction process, generating positive emotions and a tendency to forward. Consequently, consumers tended to interpret diagnostic information positively during the proposition process, leading to a positive impact on forwarding intention and changes in attitude. In contrast, when perk advertisements lacked immediate rewards, consumers’ existing negative attitudes were activated, and they tended to interpret allowance information in a cognitively consistent way to support their negative attitudes toward perk advertisements. Regardless of the level of information diagnosticity, consumers showed less forwarding behavior and smaller changes in attitude.

Existing research on advertisement attitudes has mainly focused on how advertising factors influence the generation of consumer advertisement attitudes, with less attention to the process of advertisement attitude change. Studies focusing on long-term attitude change indicate that attitudes changed through the central route are considered more persistent and impactful than those changed through peripheral routes [78]. Some scholars have looked at the immediate influence of emotions on online cognitive processing, finding that shame emotions affect consumers’ immediate cognitive processing (e.g., more advertisement memory), leading to more positive immediate advertisement attitudes [79]. The present study discovered a similar conclusion in the context of online perk advertisement—positive emotions elicited by immediate reward affected cognitive processing of information diagnosticity during propositional processing, promoting immediate attitude change.

To further validate our hypothesis, we also drew on the P2 and N2 components of event-related potentials to propose the cognitive processing process during immediate attitude change from the perspective of cognitive neuroscience. This is an innovative aspect of our research in the field of advertisement attitude change. Without immediate reward, information diagnosticity did not significantly affect P2 and N2 amplitude. With immediate reward, information diagnosticity negatively influenced P2 amplitude and positively influenced N2 amplitude. P2, as an important indicator of attentional resource allocation, reflects the impact of instant rewards on unconscious attentional allocation in cognitive processing of information diagnosticity during individuals’ immediate change of attitudes towards perk advertisement. N2 representing cognitive conflict indicates propositional conflict and trade-offs in individuals’ subsequent deeper cognitive processing [80].

### 4.1 Theoretical contributions

Firstly, this study integrates the perspectives of attitude construction and retrieval, combining the APE model and confirmation bias theory, to explore the immediate attitude change of consumers in new contexts recommended by platform precision algorithms. This deepens our understanding of immediate attitudes in the study of consumer advertising attitude formation and change.

Secondly, the study introduces the APE model, enriching theoretical perspectives on perk advertisements forwarding research. In the field of advertisement marketing, few studies focused on consumer attitudes and behavioral responses from the perspective of the APE model [54, 81]. Perk advertisements are constantly changing, and consequently, consumers’ advertising attitudes are also a dynamic development process. The APE model breaks the limitations of stable attitudes, emphasizing the internal dynamics of attitude change. It clarifies that in the decision-making process of forwarding perk advertisements, the activation of emotions initiated by immediate rewards during the association process influences proposition evaluation from the bottom-up, and the activation of persuasive knowledge during the proposition process influences associative activation from the top-down. These dynamic mechanisms affect willingness to forward perk advertisements, expanding the application boundaries of the APE model.

Thirdly, the study employs an ERPs experiment combined with self-report research, providing an effective explanation of consumers’ attitude change process toward perk advertisements through mutual verification of objective and subjective data. Previous studies only measured the change of consumers’ advertising attitude from the level of behavioral intention, but did not provide relevant evidence of cognition process [82, 83]. The organic combination of these two methods allows measuring real-time cognitive data in attitude changes via ERPs while compensating for limitations in observing subjective changes [38]. This approach enhances experimental design rigor and credibility of conclusions, providing methodology insights for future related studies.

### 4.2 Management implications

Firstly, Platforms exploiting consumers’ confirmation biases to induce immediate positive emotions and guide their sharing behavior of perk advertisements constitutes cognitive manipulation of consumers [84]. As persuasive knowledge development has a lag, consumers’ awareness will eventually lead to resistance to perk advertisements, negative platform reputation, and even user uninstallations. Such unethical marketing practices are unsustainable. Platforms should respect consumer autonomy, adhere to business ethics, promote ethical technology, and establish a virtuous cycle for platform development to avoid sacrificing ethical principles for short-term gains.

Secondly, governments should strengthen platform supervision, promptly curb boundary-pushing behaviors by platforms that manipulate consumers and infringe on their autonomy, provide value guidance for the direction of technological development, and enact appropriate regulations. This will foster a healthy internet ecosystem and better protect consumer interests.

### 4.3 Research limitations

This study aimed to uncover the internal cognitive mechanisms and real decision responses of consumers in the process of forwarding perk advertisements. However, due to limitations in the participants and experimental methods, there are still shortcomings. The study included participants from a homogeneous group of university students, which may reduce the external validity of the research. The limited sample diversity raises questions about whether consumers from different professions, age groups, and personality traits exhibit the same psychological mechanisms and behavioral responses. Future research can broaden the participant pool to include more diverse demographics to validate the robustness of the research findings.

This study was conducted entirely in a laboratory setting, with manipulation of key variables to simulate consumers’ immediate decision-making scenarios. However, because many irrelevant factors were controlled, there may be deviations between the simulated environment and real-life situations. Future research could employ mixed methods, such as field experiments, to investigate these phenomena in real-world contexts, providing more comprehensive empirical data to support the research conclusions.
